# Sorting out the mix in microbial genomics

**DOI:** 10.1111/j.1462-2920.2008.01811.x

**Published:** 2008-12

**Authors:** Michael Y Galperin

**Affiliations:** National Center for Biotechnology Information, National Library of Medicine, National Institutes of HealthBethesda, MD 20894, USA

The relatively small number of microbial genomes completed in the past two months (Table 1) includes, however, representatives of two new bacterial phyla, *Dictyoglomi* and *Nitrospirae*. To highlight the first genome sequences from these poorly studied taxa, they have been placed in a new section at the top of Table 1.

**Table 1:** Recently completed microbial genomes (August–September 2008).

Species name	Taxonomy	GenBank accession	Genome size (bp)	Proteins (total)	Sequencing centre^a^	Reference
New taxa						
*Dictyoglomus thermophilum*	*Dictyoglomi*	CP001146	1 959 987	1912	TIGR/JCVI	Unpublished
*Thermodesulfovibrio yellowstonii*	*Nitrospirae*	CP001147	2 003 803	2033	TIGR/JCVI	Unpublished
New organisms						
*Plasmodium knowlesi*	*Eukaryota, Apicomplexa*	AM910983–AM910996	23.5 Mbp	5188	Sanger institute	Pain *et al.* (2008)
*Plasmodium vivax*	*Eukaryota*, *Apicomplexa*	AAKM00000000	26.8 Mbp	5433	TIGR/JCVI	Carlton *et al.* (2008)
*Coprothermobacter proteolyticus*	*Firmicutes*	CP001145	1 424 912	1482	JCVI	Unpublished
*Streptococcus equi*	*Firmicutes*	CP001129	2 024 171	1893	Methodist hospital	Beres *et al.* (2008)
*Phenylobacterium zucineum*	*α-Proteobacteria*	CP000747, CP000748	3 996 255 382 976	3854	Zhejiang University	Luo *et al.* (2008)
*Acidithiobacillus ferrooxidans* ATCC 23270	*γ-Proteobacteria*		2 982 397	3217	TIGR/JCVI	Valdés *et al*. (2008)
*Acidithiobacillus ferrooxidans* ATCC 53993	*γ-Proteobacteria*	CP001132	2 885 038	2826	JGI	Unpublished
*Alteromonas macleodii* ‘Deep ecotype’	*γ-Proteobacteria*	CP001103	4 412 282	4072	JCVI	Unpublished
*Anaeromyxobacter* sp. K	*δ-Proteobacteria*	CP001131	5 061 632	4457	JGI	Unpublished
*Geobacter bemidjiensis*	*δ-Proteobacteria*	CP001124	4 615 150	4018	JGI	Unpublished
*Borrelia duttonii*	*Spirochaetes*	CP000976–CP000992	1.57 (total)	1305	Genoscope	Lescot *et al.* (2008)
*Borrelia recurrentis*	*Spirochaetes*	CP000993–CP001000	1.24 (total)	990	Genoscope	Lescot *et al.* (2008)
New strains						
*Klebsiella pneumoniae* 342	*γ-Proteobacteria*	CP000964, CP000965, CP000966	5 641 239 187 922 91 096	5768	JCVI	Fouts *et al*. (2008)
*Salmonella enterica* ssp. *enterica* serovar Agona str. SL483	*γ-Proteobacteria*	CP001138, CP001137	4 798 660 37 978	4614	JCVI	Unpublished
*Salmonella enterica* ssp. *enterica* serovar Dublin str. CT_02021853	*γ-Proteobacteria*	CP001144, CP001143	4 842 908 74 551	4617	JCVI	Unpublished
*Salmonella enterica* ssp. *enterica* serovar Gallinarum str. 287/91	*γ-Proteobacteria*	AM933173	4 658 697	3965	Sanger Institute	Thomson *et al*. (2008)
*Salmonella enterica* ssp. *enterica* serovar Enteritidis str. P125109	*γ-Proteobacteria*	AM933172	4 685 848	4206	Sanger Institute	Thomson *et al.* (2008)
*Salmonella enterica* ssp. *enterica* serovar Paratyphi A str. AKU_12601	*γ-Proteobacteria*	FM200053	4 581 797	4078	Sanger Institute	Unpublished
*Aliivibrio (Vibrio) fischeri*MJ11	*γ-Proteobacteria*	CP001139, CP001133, CP001134	2 905 029 1 418 848 179 459	4039	JCVI	Unpublished
*Helicobacter pylori*G27	*ε-Proteobacteria*	CP001173, CP001174	1 652 982 10 031	1504	University of Oregon	Unpublished

a Sequencing centre names are abbreviated as follows: Genoscope, Centre National de Séquençage, Evry, France; JCVI, J. Craig Venter Institute, Rockville, Maryland, USA; JGI, US Department of Energy Joint Genome Institute, Walnut Creek, California, USA; Methodist Hospital, Center for Molecular and Translational Human Infectious Disease Research, The Methodist Hospital Research Institute, Houston, Texas, USA; Sanger Institute, The Wellcome Trust Sanger Institute, Wellcome Trust Genome Campus, Hinxton, Cambridgeshire, UK; TIGR, The Institute for Genomic Research (currently J. Craig Venter Institute), Rockville, Maryland, USA; University of Oregon, Institute of Molecular Biology, University of Oregon, Eugene, Oregon, USA; Zhejiang University, Cancer Institute, The Second Affiliated Hospital, Zhejiang University Medical School, Hangzhou, Zhejiang, China.

So far, little is known about either *Dictyoglomus thermophilum* or *Thermodesulfovibrio yellowstonii.* Both are Gram-negative thermophilic heterotrophs with an extremely low (29 mol%) G+C content of their chromosomal DNA (Saiki *et al.*, 1985; Henry *et al.*, 1994). *Dictyoglomus thermophilum* is an obligate anaerobe that grows optimally at 70–73°C. It was isolated from the Tsuetate hot spring in Japan (Saiki *et al.*, 1985) and used to purify three extremely heat-stable amylases (Horinouchi *et al.*, 1988). *Thermodesulfovibrio yellowstonii* was isolated from a thermal vent in Yellowstone Lake in Wyoming. It contains *c*-type cytochromes and grows optimally at 65°C using lactate, pyruvate or formate plus acetate as substrates and can use sulfate, thiosulfate and sulfite as terminal electron acceptors (Henry *et al.*, 1994). Analysis of these genomes should provide a window into the physiology and evolutionary relationships of these new bacterial lineages.

Completion of these two genomes is a major step towards the goal of having at least one complete genome sequence from representatives of all major prokaryotic groups. Indeed, we now have at least one completely sequenced genome for 18 bacterial phyla out of the 24 listed in the taxonomic outline in the socond volume of the *Bergey's Manual* (Garrity *et al*., 2004; available at http://www.bergeys.org/outlines.html). Representatives of five more bacterial phyla are at various stages of genome sequencing: *Chrysiogenes arsenatis* (phylum *Chrysiogenetes*), *Denitrovibrio acetiphilus* (*Deferribacteres*), *Fibrobacter succinogenes* (*Fibrobacteres*), *Thermodesulfobacterium commune* (*Thermodesulfobacteria*) and *Thermomicrobium roseum* (*Thermomicrobia*, which may be considered a class in the phylum *Chloroflexi*). Only one of those 24 phyla (*Gemmatimonadetes*) remains not a subject of any publicly announced genome sequencing project. This is obvious progress in coverage of microbial diversity compared with the status of the genome sequencing just two years ago (Galperin, 2006),

However, this nice and clear picture of the bacterial taxonomy and the corresponding genome sequencing efforts is complicated by several different factors. First of all, high-rank bacterial taxonomy is still in the state of flux: new candidate phyla are being identified and new genome sequencing projects are being planned to characterize their representatives. There are ongoing sequencing projects for *Lentisphaera araneosa* and *Thermanaerovibrio acidaminovorans*, cultured representatives, respectively, of the recently recognized phyla *Lentisphaerae* (Cho *et al*., 2004) and *Synergistetes* (*Aminanaerobia*) (Hongoh *et al*., 2007; Jumas-Bilak *et al*., 2007). In addition, genomic sequencing is being performed on candidate phyla that were initially deduced based solely on the clustering of 16S rRNA sequences. Examples include a nearly complete genome from the candidate phylum TM7, which still has no cultivated members (Marcy *et al*., 2007) and two recently sequenced genomes from representatives of the candidate phylum Termite group 1 (TG1), one of which, “*Elusimicrobium minutum*”, was in the meantime successfully cultivated. Sometimes genomic data reveal distant similarities between two or more phyla, which results in their unification into a group (e.g. *Bacteroidetes*/*Chlorobi*, *Fibrobacteres*/*Acidobacteria*) or a superphylum, e.g. *Chlamydiae*/*Verrucomicrobia*/*Planctomycetes*/*Lentisphaerae* (Wagner and Horn, 2006; Hou *et al*., 2008). Besides, certain validly described bacterial groups still lack any sequence information (Yarza *et al*., 2008). Finally, there are several alternative classifications of bacteria that made their way into taxonomic literature but, for a variety of reasons, failed to gain acceptance in the community (Gupta, 1998; 2000; Cavalier-Smith, 2002; 2006). Another such example is the already mentioned (Galperin, 2008) recent transfer of *Mollicutes* from the phylum *Firmicutes* into a new phylum *Tenericutes* in the latest edition of *Bergey's Manual* (Ludwig *et al*., 2008). The phylogenetic trees that served as the rationale for that move show numerous inconsistencies and hardly justify the decision to create this new phylum.

It must be noted that back in 1992, Sneath and Brenner stated ‘There is no such thing as an official classification’ (see http://www.bacterio.cict.fr/Sneath-Brenner.html). This point was recently reiterated by J.P. Euzéby, whose List of Prokaryotic names with Standing in Nomenclature (http://www.bacterio.cict.fr/) includes an up-to-date listing of commonly recognized prokaryotic phyla (http://www.bacterio.cict.fr/classifphyla.html). For a quick look at the current state of microbial genome sequencing, the easiest tool might be the NCBI's Tax Tree (http://www.ncbi.nlm.nih.gov/genomes/MICROBES/microbial_taxtree.html), which lists both completed and ongoing genome sequencing projects. However, for those interested in the emerging microbial diversity, the best source of information is probably the ‘greengenes’ website (http://greengenes.lbl.gov), which includes six different classification schemes, from the most conservative ones (the Ribosomal Database project) to the schemes by Pace and Hugenholtz that include up to 100 bacterial lineages (see http://greengenes.lbl.gov/Download/Taxonomic_Outlines/five_way_venn.png for a nice graphical representation of the relation between these schemes).

Among archaea, Bergey's taxonomic outline recognized two phyla, *Crenarchaeota* and *Euryarchaeota*, which are now represented by 50 complete genomes (16 and 34 respectively). In addition, there are two newly suggested but not yet officially recognized phyla, each represented by a single genome, “*Candidatus* Korarchaeum cryptofilum” (*Korarchaeota*) and *Nanoarchaeum equitans* (*Nanoarchaeota*). Again, classifications by Pace and Hugenholtz at http://greengenes.lbl.gov include up to 40 archaeal lineages.

Among eukaryotic microorganisms, few genomes have been sequenced to any significant degree, and most of these genomes represent just a handful of lineages: *Amoebozoa* (*Dictyostelium, Entamoeba*), *Apicomplexans* (*Cryptosporidium*, *Plasmodium*, *Toxoplasma*), *Ciliophora* (*Paramecium*, *Tetrahymena*) and *Kinetoplasts* (*Leishmania*, *Trypanosoma*). There are partially sequenced representatives of choanoflagellates (*Monosiga*), diplomonads (*Giardia*), parabasalids (*Trichomonas*), rhodophytes (*Cyanidioschyzon*) and stramenopiles (*Phytophtora*, *Thalassiosira*). Several more eukaryotic lineages (*Apusozoa*, *Haptophyceae Heterolobosea*) are going to be covered by ongoing sequencing projects, but the overall coverage of major eukaryotic groups is extremely poor and will remain that way at least for the next several years.

In summary, recent genomic projects are successfully covering the diversity of cultured bacteria and archaea. Any significant coverage of the main eukaryotic lineages or of prokaryotic lineages identified through cultivation-independent methods will remain the challenge for years to come.

In eukaryotic genomics, the biggest news was the completion of the genome sequences of two malaria parasites, *Plasmodium knowlesi* and *Plasmodium vivax.* Their back-to-back publication in *Nature* (Carlton *et al*., 2008; Pain *et al*., 2008) was accompanied by an excellent commentary (Winzeler, 2008) on the contribution of the genomic data to the progress of malaria research in the 6 years that have passed since the completion of the genomes of *Plasmodium falciparum* (Gardner *et al*., 2002) and *Plasmodium yoelii* (Carlton *et al*., 2002).

*Coprothermobacter proteolyticus* (formerly known as *Thermobacteroides proteolyticus*) is a moderately thermophilic proteolytic bacterium, originally isolated from the fermentation sludge of an anaerobic digester treating cattle manure mixed with tannery waste (Ollivier *et al*., 1985). It can grow at temperatures of up to 75°C with an optimum at 63°C. Although *C. proteolyticus* was initially described as a Gram-negative bacterium and therefore suggested to belong to a deep bacterial lineage, potentially at the phylum level (Rainey and Stackebrandt, 1993), analysis of its 16S rRNA revealed that it is related to *Thermoanaerobacter* sp. It is currently assigned to the family *Thermodesulfobiaceae* (Mori *et al*., 2003) within the order *Thermoanaerobacterales* and is the first sequenced genome from that family.

*Phenylobacterium zucineum* is an α-proteobacterium recently isolated from a human erythroleukemia cell line (Zhang *et al*., 2007). All close relatives of *P. zucineum* are free-living environmental organisms, and its 4.4 Mb genome is much larger than that of any intracellular parasite or symbiont characterized so far. Indeed, the genome sequence (Luo *et al*., 2008) revealed similarities with the genome of *Caulobacter crescentus*. However, fragments of *P. zucineum* genomic DNA were found among the EST libraries from breast cancer and lymphatic cell lines, suggesting that this organism might survive in proliferative tissues.

*Acidithiobacillus ferrooxidans* (previously known as *Thiobacillus ferrooxidans*; Kelly and Wood, 2000), is an obligately acidophilic chemolithoautotrophic γ-proteobacterium, a popular model organism to study bacterial membrane energetics at acidic pH values (see Ferguson and Ingledew, 2008 for a recent review). It gains energy by oxidizing ferrous iron and is able to grow in the pH range from 1.3 to 4.0 using CO_2_ as the sole source of carbon. *Acidithiobacillus ferrooxidans* is a major component of microbial consortia used in bio-mining to extract copper, zinc and other metals from low-grade ores. With the recent increase in the price of gold, *A. ferrooxidans*-based microbial consortia are increasingly used to improve recovery of gold from arsenopyrite ores. Despite the importance of this organism (or maybe because of it), sequencing of the *A. ferrooxidans* genome had a long and convoluted history. The first (incomplete or ‘gapped’) genome sequence of the type strain *A. ferrooxidans* ATCC 23270 was produced at the Integrated Genomics in 1999. It consisted of 1353 contigs covering 2611 kb and coding for 2712 proteins; it was estimated to lack ∼100 kb (Selkov *et al*., 2000). This sequence was used for an analysis of the amino acid metabolism in *A. ferrooxidans*, which allowed an almost complete reconstruction of its metabolic pathways, leaving just 10 unassigned (missing) enzymes. Despite the initial intent of the authors to demonstrate that ‘gapped’ microbial genomes were almost as good as complete ones (Selkov *et al*., 2000), this paper actually succeeded in proving the opposite: a meaningful and unequivocal analysis is only possible with a complete genome sequence. Furthermore, only small pieces of the genome have been submitted to the GenBank, which prevented others from analysing this genome.

Shortly after that, sequencing of *A. ferrooxidans* genome has been undertaken at the Institute of Genomic Research (TIGR). The resulting incomplete genome sequence of 3081 kb was made publicly available in 2001 as RefSeq entry NC_002923 and was subsequently used for a variety of genome analyses (e.g. Valdés *et al*., 2003; Quatrini *et al*., 2007). Over the next two years, this sequence was updated more than a dozen times and was finally withdrawn at the end of 2003. Since 2006, a complete genome sequence of 2982 kb coding for 3217 predicted proteins has been available on the TIGR website but was not submitted to GenBank. Finally, a recent joint paper by Chilean and TIGR scientists (Valdés *et al*., 2008) reported a detailed analysis of this genome and its availability to the public.

Meanwhile, JGI scientists have released a 2885 kb genome sequence of another strain of *A. ferrooxidans*, which encodes 2826 proteins. This strain *A. ferrooxidans* ATCC 53993 was isolated from mine water of the Alaverda copper deposit in Armenia and initially assigned the name *Leptospirillum ferrooxidans* (Balashova *et al*., 1974; Hippe, 2000). Although its relation to the type strain ATCC 23270 is not known at this time, their 16S rRNA sequences are 100% identical. Thus, after 10 years of struggling with unfinished genome sequences, the public now has access to two complete genomes of *A. ferrooxidans*. This should allow further analyses of the properties of this remarkable organism and stimulate its use in energy research and bio-mining.

The list of completely sequenced spirochaete genomes has grown to include genomes of *Borrelia duttonii* and *Borrelia recurrentis* (Lescot *et al*., 2008). Both organisms are important human pathogens causing relapsing fevers. The first one is transmitted by the tick *Ornithodoros moubata* and is found primarily in east Africa. *Borrelia recurrentis* is transmitted by human body lice *Pediculus humanus* and is found in around the world. The sequenced strain *B. duttonii* Ly was isolated from a 2-year-old girl with tick-borne relapsing fever in Tanzania, whereas *B. recurrentis* strain A1 was isolated from an adult patient with louse-borne relapsing fever in Ethiopia.

*Klebsiella pneumoniae* ssp. *pneumoniae* is a well-known human pathogen, and the first genome of its clinical isolate MGH 78578 was sequenced more than two years ago. A very interesting paper from the JCVI scientists now reports the genome sequence of an environmental N_2_-fixing strain of *K. pneumoniae* (Fouts *et al*., 2008). Such strains are commonly found as endophytes that colonize tissues of rice, maize, sugarcane, banana and various grasses and improve the growth of the host plants by supply them with ammonia. The sequenced strain *K. pneumoniae* 342 was isolated from maize and later shown to colonize wheat and alfalfa sprouts. Comparative analysis of the two strains provides interesting clues to the adaptation to the endophytic lifestyle, as well as into the evolution of pathogenicity in *K. pneumoniae*.

The list of organisms with recently sequenced genomes also includes the marine γ-proteobacteria *Alteromonas macleodii* and *Vibrio fischeri*, δ-proteobacteria *Anaeromyxobacter* sp. and *Geobacter bemidjiensis*, five new strains of *Salmonella enterica* ssp. *enterica* that include four new serovars (Thomson *et al*., 2008), *Streptococcus equi* ssp. *zooepidemicus* (also known as *Streptococcus zooepidemicus*), the cause of an acute nephritis acute epidemic in Brazil (Beres *et al*., 2008), and the well-studied *Helicobacter pylori* strain G27 (Table 1).
